# A new synthetic matrix metalloproteinase inhibitor reduces human mesenchymal stem cell adipogenesis

**DOI:** 10.1371/journal.pone.0172925

**Published:** 2017-02-24

**Authors:** Dale B. Bosco, Mark D. Roycik, Yonghao Jin, Martin A. Schwartz, Ty J. Lively, Diego A. R. Zorio, Qing-Xiang Amy Sang

**Affiliations:** 1 Institute of Molecular Biophysics, Florida State University, Tallahassee, Florida, United States of America; 2 Department of Chemistry & Biochemistry, Florida State University, Tallahassee, Florida, United States of America; University of South Alabama, UNITED STATES

## Abstract

Development of adipose tissue requires the differentiation of less specialized cells, such as human mesenchymal stem cells (hMSCs), into adipocytes. Since matrix metalloproteinases (MMPs) play critical roles in the cell differentiation process, we conducted investigations to determine if a novel mercaptosulfonamide-based MMP inhibitor (MMPI), YHJ-7-52, could affect hMSC adipogenic differentiation and lipid accumulation. Enzyme inhibition assays, adipogenic differentiation experiments, and quantitative PCR methods were employed to characterize this inhibitor and determine its effect upon adipogenesis. YHJ-7-52 reduced lipid accumulation in differentiated cells by comparable amounts as a potent hydroxamate MMPI, GM6001. However, YHJ-7-82, a non-inhibitory structural analog of YHJ-7-52, in which the zinc-binding thiol group is replaced by a hydroxyl group, had no effect on adipogenesis. The two MMPIs (YHJ-7-52 and GM6001) were also as effective in reducing lipid accumulation in differentiated cells as T0070907, an antagonist of peroxisome-proliferator activated receptor gamma (PPAR-gamma), at a similar concentration. PPAR-gamma is a typical adipogenic marker and a key regulatory protein for the transition of preadiopocyte to adipocyte. Moreover, MMP inhibition was able to suppress lipid accumulation in cells co-treated with Troglitazone, a PPAR-gamma agonist. Our results indicate that MMP inhibitors may be used as molecular tools for adipogenesis and obesity treatment research.

## Introduction

Obesity is an epidemic affecting one-third of Americans [1, 2]. This disorder is a major risk factor for numerous conditions, including cardiovascular disease, stroke, diabetes, and cancer [3–6]. Associated with obesity is adipocyte hypertrophy and hyperplasia, the latter being the direct consequence of increased adipogenic stimulation. It was originally assumed that adipocyte number rarely changed post adolescence. However, it is now known that approximately 10% of the adult body’s adipocytes are replaced annually, highlighting the importance of adipogenesis [7].

The most commonly studied *in vitro* model of adipogenesis is the murine-derived 3T3-L1 cell line, first isolated by Green and Kehinde in 1974 [8]. These cells can be directed to accumulate lipids and adopt an adipocyte-like phenotype [9]. Human mesenchymal stem cells (hMSCs) also differentiate into adipocytes, and represent another useful model of adipogenesis [10]. It should be noted however, that unlike 3T3-L1 cells, hMSCs do not require a mitotic clonal expansion phase before adipogenesis is initiated [10, 11]. Moreover, the differentiation of hMSCs into mature adipocytes occurs in two phases; a determination phase where hMSCs commit to adipogenesis and become pre-adipocytes, and a differentiation phase where pre-adipocytes, in a manner similar to 3T3-L1 cells, begin secreting adipocyte-specific proteins and accumulate lipids [11, 12].

Although adipogenesis follows a relatively discrete series of events, it remains strongly influenced by extracellular factors, principally the extracellular matrix (ECM). During adipogenic differentiation of the pre-adipocyte, fibronectin-rich ECM is converted to a looser adipocyte basement membrane, suggesting direct involvement of matrix metalloproteinases (MMPs), a family of enzymes that utilized catalytically active zinc(II) to hydrolyze and modulate almost every ECM component [13]. Following the discovery of MMPs, numerous synthetic inhibitors have been created that incorporate a variety of zinc-binding groups (ZBGs) including hydroxamates, carboxylates, and phosphinyls [14–15]. The most potent matrix metalloproteinase inhibitors (MMPIs) utilize hydroxamates, which coordinate Zn^2+^ in a bidentate fashion [15, 16]. However, the affinity for Zn^2+^ displayed by hydroxamate ZBGs tends to overwhelm proteinase specificity and lead to target promiscuity. As a result, our group focused on developing MMPIs that contain the less implemented mercaptan ZBG [17–26]. The current YHJ series of MMPIs incorporates a mercaptosulfonamide attached to a diphenyl ether to increase selectivity between MMPs based on S1’ pocket depth [25–28]. Our latest inhibitors also display potencies similar to hydroxamate MMPIs for a number of MMPs [26]. Consequently, we sought to determine if one of our most promising inhibitors, YHJ-7-52, could affect the adipogenic differentiation of hMSCs.

## Materials and methods

### Materials

The mercaptosulfide matrix metalloproteinase (MMP) inhibitor, YHJ-7-52, and its structural control, YHJ-7-82, were designed and synthesized by Drs. Martin A. Schwartz and Yonghao Jin as described [24, 26]. GM6001 was synthesized by Dr. Yonghao Jin. Troglitazone and T0070907 were purchased from Cayman Chemical Co. (Ann Arbor, MI). The adult human mesenchymal stem cell (hMSC) line was obtained from the Tulane University Center of Gene Therapy (New Orleans, LA). Human fibroblast collagenase (MMP-1) and gelatinase A (MMP-2) were provided by Dr. L. Jack Windsor (Indiana University, Indianapolis, IN) [27], recombinant human matrilysin-1 (MMP-7) was provided by Dr. Harold E. Van Wart (Syntex, Palo Alto, CA) [28], human neutrophil gelatinase B was purified from human blood by Dr. Qing-Xiang Sang as described previously [29], and human recombinant membrane-type 1 matrix metalloproteinase (MT1-MMP, MMP-14) was provided by Dr. Harald Tscheche (Bielefeld University, Bielefeld, Germany) [22–23]. All other commonly used reagents and materials were obtained from either VWR (Radnor, PA) or Sigma Aldrich (St. Louis, MO) unless otherwise specified.

### Enzyme inhibition assay

Enzymatic assays to characterize inhibitor potency were performed as previously described [22, 30]. Assays were performed at 25°C in 50 mM 4-(2-hydroxyethyl)-1-piperazineethanesulfonic acid (HEPES) buffer at pH 7.5 with 10 mM CaCl_2_, 200 m*M* NaCl, 0.01% (w/v) Brij-35, and 5 *μ*M tris(2-carboxyethyl)-1-piperazineethanesulfonic acid (TCEP). The fluorogenic substrate Mca-PLGLDpa-AR-NH_2_ (M-1895, Bachem, Torrance, CA) was used at a concentration of 1 *μ*M as the substrate to measure inhibition constants. Briefly, 10 *μ*L of inhibitor solution or control (100% DMSO), 176 *μ*L of assay buffer, and 10 *μ*L of enzyme solution were mixed and incubated for 30 min prior to initiation of the assay, which was accomplished by adding 4 *μ*L of substrate solution, 5% final concentration DMSO. The release of product was monitored by fluorescence (λ_ex_ = 328 nm, λ_em_ = 393 nm) with a PerkinElmer luminescence spectrophotometer LS50B (FL WinLab v3.0) connected to a temperature-controlled water bath. Inhibitor potency was determined *via* comparison of relative rates (v_i_/v_o_).

### Mesenchymal stem cell culture

Low passage human mesenchymal stem cells (hMSCs) were cultured in alpha-modified minimum essential medium (αMEM) supplemented with an antibiotic cocktail composed of 10,000 IU penicillin and 10 mg/mL streptomycin, plus 200 mM L-glutamine, and 20% fetal bovine serum (FBS; GE Healthcare, Picataway, NJ). Prior to experimentation hMSCs were plated for 24 h of recovery and maintained at 37°C in a humidified environment containing 5% CO_2_. Cells were then seeded into appropriate culture ware, with media changed every 72 h until the desired confluence was reached.

### Cytotoxicity assay

Mercaptosulfonamide MMPIs were dissolved in 100% DMSO to generate 8 mM stock solutions and subsequently diluted with αMEM, while maintaining the final DMSO concentration at 1.25% (v/v). Media supplemented with a panel of final inhibitor concentrations ranging from 0–100 *μ*M were then added to 70–80% confluent hMSC cultures and incubated for 24 h. At the end of treatment, inhibitor-conditioned media was removed and cells were washed with phosphate buffered saline (PBS) (pH 7.4) prior to being trypsinized (0.25% Trypsin, GE Healthcare), pelleted, and resuspended in 1 mL of fresh αMEM. Aliquots of the cell suspension for each condition were diluted 1:1 with 0.4% (w/v) Trypan Blue and counted with a hemocytometer. Relative cytotoxicity was determined by normalizing cell number for each condition against its respective control. A concentration was arbitrarily deemed cytotoxic if it produced a statistically significant reduction in cell number when compared to DMSO controls.

### Induction of adipogenesis

Human MSCs were seeded into 6-well plates at 1,000 cells/cm^2^_._ Upon reaching 70–80% confluence hMSCs were induced towards an adipogenic lineage by adding dexamethasone (0.5 *μ*M), 3-isobutyl-1-methylxanthine (0.5 *μ*M), and indomethacin (50 *μ*M) to the culture medium [31]. Tested compounds were dissolved in DMSO and added to treatment wells during differentiation to a final total concentration of 10 *μ*M with DMSO concentrations remaining constant (0.125% or 0.25% v/v) between conditions. Wells treated with appropriate concentrations of DMSO alone served as controls. Media and treatments were refreshed every 72 h for 21 days.

### Cell staining and quantification of lipid accumulation

After 21 days of differentiation, hMSCs were washed with PBS and fixed with neutral buffered formalin for 1 h. Cells were then stained with 0.3% (w/v) Oil Red-O in 3:2 100% isopropyl alcohol to PBS for 20 min, washed with PBS, stained with 5 *μ*g/mL Hoechst 33342 for 5 min, then washed again. Cultures were later imaged with an epifluorescence capable Nikon TE2000-E2 Eclipse microscope. Relative staining was determined with ImageJ (version 1.46c, National Institutes of Health, Bethesda, MD) by standardizing the area stained in each image to nuclei number prior to normalizing against DMSO treated controls.

### Quantitative real-time polymerase chain reaction (qPCR)

MMPI treated and untreated hMSCs were collected every 72 h over 21 days of differentiation. Total RNA from each time point was isolated using the E.Z.N.A. Total RNA Kit I (Omega Bio-Tek, Norcross, GA) and purified with the DNA-free RNA Kit (Zymo Research, Irvine, CA). RNA was resuspended in nuclease-free water, with concentrations determined *via* a Nanodrop UV-Vis Spectrophotometer (ThermoFisher Scientific, Waltham, MA). 500 ng of RNA was used for cDNA synthesis using qScript cDNA SuperMix (Quanta Bioscience, Gaitherburg, MD). 100 ng/*μ*L cDNA was used as template for quantitative PCR on a 7500 Fast Real Time PCR system (Life Technologies, Carlsbad, CA) using PerfeCTa SYBR® Green SuperMix, Low Rox (Quanta Bioscience). Relative gene expression was determined via the 2^^(-ΔΔCt)^ method [32]. Expression levels of PPARγ (peroxisome-proliferator activated receptor gamma) were determined. β-Actin was used as an endogenous control. Primers were designed with Primer-BLAST (NCBI) and purchased from Integrated DNA Technologies (IDT, Coralville, IA). MMP-2: forward: 5’-CATCGCTCAGATCCGTGGTG-3’ reverse: 5’-GCATCAATCTTTTCCGGGAGC-3’; MMP-14: forward: 5’-GGCTGCCTACCGACAAGATT-3’ Reverse: 5’-GTACTCGCTATCCACTGCCC-3’; PPARγ: forward: 5’-CTTGCAGTGGGGATGTCTCAT-3’ reverse: 5’-AGGTCAGCGGACTCTGGATT-3’; β-Actin: forward: 5’-AGTCCTGTGGCATCCACGAAACTA-3’ reverse: 5’-ACTCCTGCTTGCTGATCCACATCT-3’.

### Statistical analysis

All cellular and qPCR experiments described above were performed with at least three independent replicates per data point and results are expressed as the mean ± standard error. Kinetics results were repeated with three independent replicates and expressed as the mean ± standard error. Statistical analyses were performed with the least significant difference correction for one-way analysis of variance (ANOVA) for multiple comparisons followed by Tukey contrast post-hoc analysis, with statistically significant values defined as *P* < 0.05.

## Results

### YHJ-7-52 is a potent MMP-2 and MMP-14 inhibitor

Fluorogenic substrate-based enzyme inhibition assays were performed to determine IC_50_ values for the matrix metalloproteinase inhibitor (MMPI) YHJ-7-52. The potent broad-spectrum hydroxamate MMPI, GM6001, was used for comparison. The structural analog of YHJ-7-52, YHJ-7-82, was used as a negative control. YHJ-7-82 replaces the inhibitory thiol moiety with a non-inhibitory hydroxyl group. Compounds were evaluated for their potencies against a panel of MMPs relevant to adipogenesis [33]. Results are summarized in Table 1. YHJ-7-52 was highly potent for all deep and intermediate pocket MMPs, especially MMP-2 (gelatinase A) and MMP-14 (membrane-type 1 matrix metalloproteinase, MT1-MMP) where IC_50_ values were less than 6 nM. GM6001 was highly potent against every MMP tested with IC_50_ values equal to or less than 2 nM. The negative control compound YHJ-7-82 had almost undetectable inhibitory activity under the experimental conditions, indicating that the effectiveness of YHJ-7-52 is directly related to its zinc chelating group.

**Table 1:** IC_50_ values for the mercaptosulfonamide MMP inhibitor YHJ-7-52, its negative control compound YHJ-7-82, and GM6001.

	IC_50_ (nM)				
	MMP-1	MMP-2	MMP-7	MMP-9	MMP-14
**YHJ-7-52**	1200 ± 370	5.5 ± 4.2	2400 ± 720	87 ± 9	4.3 ± 0.7
**YHJ-7-82**	>200K	>100K	>200K	>200K	>200K
**GM6001**	0.4 ± 0.3	0.2 ± 0.1	2.0 ± 1.9	0.1 ± 0.1	0.5 ± 0.1

Enzyme inhibition kinetic assays were performed at 25°C in HEPES assay buffer containing 0.01% (w/v) Brij-35, and 5 *μ*M TCEP. A fluorogenic substrate was used to measure MMP hydrolytic activity. The release of product was monitored by fluorescence. Inhibitor potency (IC_50_ value) was determined *via* comparison of relative rates (v_i_/v_o_). MMP–matrix metalloproteinase.

### YHJ-7-52 is effective in reducing lipid accumulation within cellular models of adipogenesis

**Fig 1:**
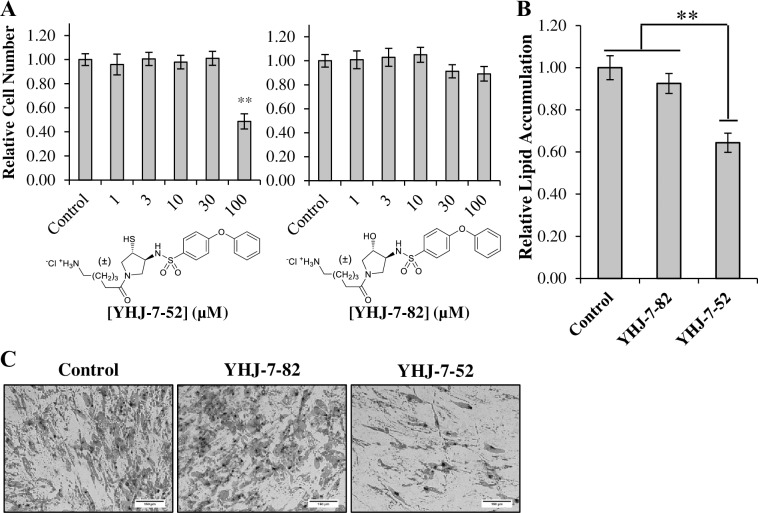
Assessment of YHJ-7-52 biological activities in hMSC culture models. (A) Human MSCs were incubated for 24 h with YHJ-7-52 and YHJ-7-82 at various concentrations (0–100 *μ*M) and then counted. Human MSC were induced towards an adipogenic lineage in the presence of either 10 *μ*M YHJ-7-52, 10 *μ*M YHJ-7-82, or DMSO control. (B) Relative lipid accumulation as determined by imageJ. YHJ-7-52 reduced lipid accumulation by 36%. (C) Representative images of each condition. Results are displayed as mean ± standard error. Scale bar 150 μm. ** *P* < 0.05

To begin our investigation we examined if YHJ-7-52 and YHJ-7-82 displayed any cytotoxic effects. Human MSCs were treated with 0–100 *μ*M of compound for 24 hours, stained with Trypan Blue, and live cells were counted. Fig 1A shows that YHJ-7-52 only produced a significant reduction in cell number at 100 *μ*M, while its non-inhibitory analog, YHJ-7-82, showed minimal cytotoxicity across all tested concentrations. Based on the cytotoxicity results, a compound concentration of 10 *μ*M was utilized for further examinations.

Next, we determined if YHJ-7-52 affected hMSC adipogenesis. Human MSCs were induced towards an adipogenic lineage in the presences of either YHJ-7-52, YHJ-7-82, or DMSO control. After 21 days differentiation was assessed by staining cells with Oil Red-O, and determining relative area of staining standardized to nuclei counts. Fig 1B and 1C show that treatment with YHJ-7-52 reduced lipid accumulation by approximately 36%, while YHJ-7-82 did not produce a significant response as anticipated.

### Gene expression levels of an adipogenic marker and two matrix metalloproteinases within human mesenchymal stem cells undergoing adipogenesis

Since image analysis only quantifies the extent of adipogenesis *via* measurements of lipid accumulation, quantitative real-time polymerase chain reaction (qPCR) was used to determine the effect of inhibitor treatment upon the transcriptional processes of hMSCs undergoing adipogenesis. For this experiment, the typical adipogenic marker, and a key regulator of adipogenesis, peroxisome-proliferator activated receptor gamma (PPARγ) was selected [34]. MMPs -2 and -14 (MT1-MMP) were selected because previous reports indicated they play principal roles in adipogenesis [33, 35].

**Fig 2:**
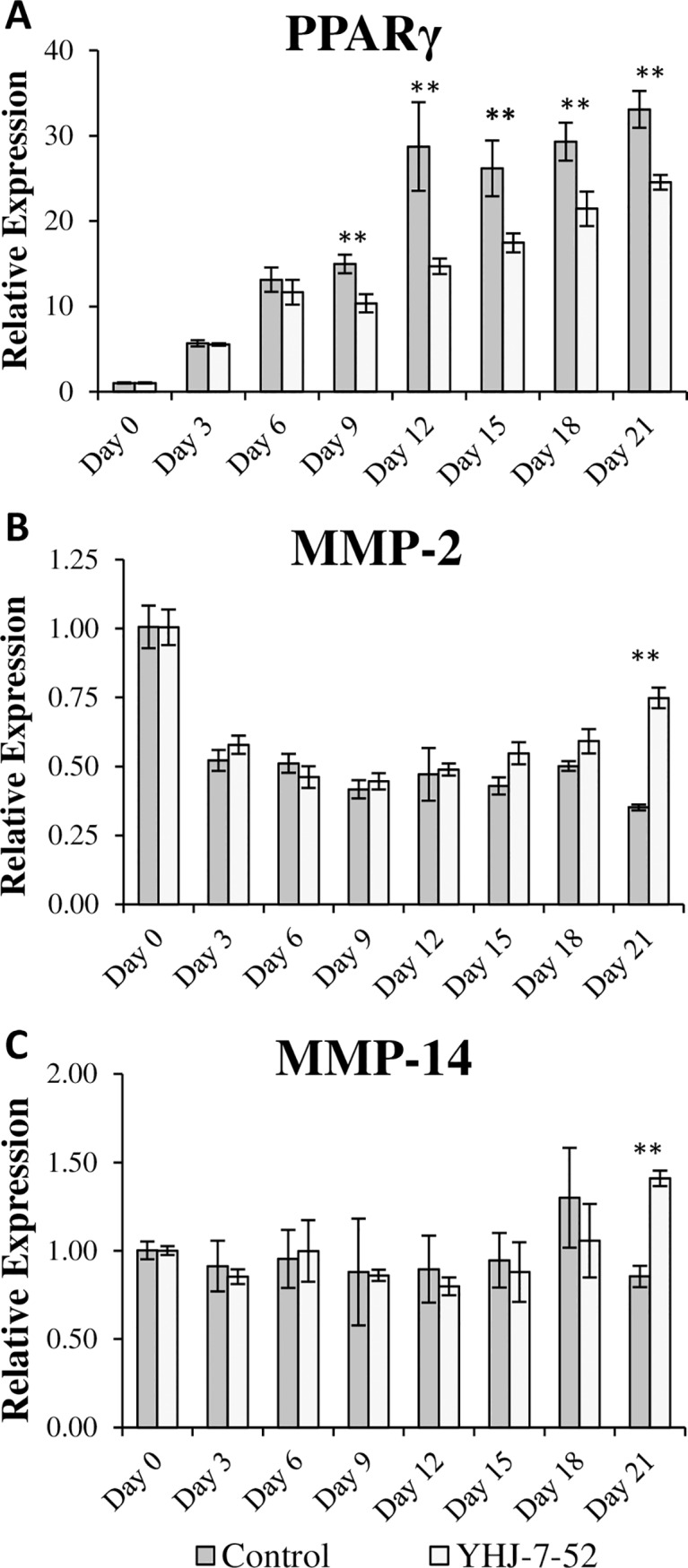
Quantitative real-time PCR to measure gene expression levels of select adipogenic markers and MMPs in hMSCs induced toward an adipogenic linage. Human MSCs were treated with either YHJ-7-52 (10 *μ*M) or DMSO control. Expression of (A) PPARγ, (B) MMP-2, and (C) MMP-14 over time. Results are displayed as mean ± standard error. ** *P* < 0.05

Human mesenchymal stem cells (hMSCs) were induced to undergo adipogenic differentiation in the presence of YHJ-7-52 for 21 days with cells harvested every 72 h. Fig 2A shows treatment with YHJ-7-52 significantly suppressed PPARγ expression after day 9 of treatment when compared to DMSO controls. This indicates that YHJ-7-52 is influencing not only lipid accumulation but also differentiation. MMP-2 gene expression level was uniformly reduced after induction of adipogenesis by approximately 50%, while MMP-14 gene expression level remained relatively consistent (Fig 2B and 2C). These results indicate that YHJ-7-52 does not affect MMP-2 and MMP-14 gene expression levels. For unknown reasons, on Day 21 upon the maturation of the adipocytes, the gene expression levels of MMP-2 and MMP-14 are significantly higher in the presence of YHJ-7-52 compared to the negative control.

### MMP inhibition reduces lipid accumulation in the presence of a peroxisome proliferator activated receptor gamma antagonist or agonist

**Fig 3:**
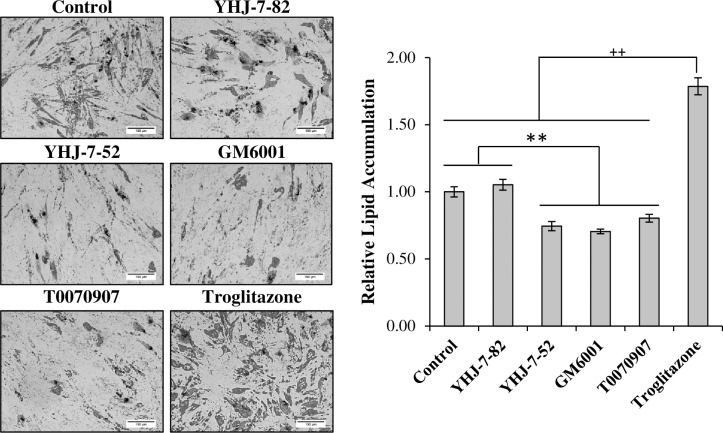
Comparison of the effect of MMPIs and PPARγ modulators on adipogenesis. Human MSCs undergoing adipogenic differentiation were cultured in the presence of either YHJ-7-82, YHJ-7-52, GM6001, T0070907, Troglitazone, or DMSO control. Final concentration of all compounds used was 10 *μ*M. Left, representative images detailing Oil Red-O staining. Right, inageJ analysis results. Results are displayed as mean ± standard error. Scale bar 150 μm. ** *P* < 0.05, ++ *P* < 0.05.

Our results indicate that MMPI treatment reduces the adipogenic differentiation of hMSCs. Consequently, we wanted to determine if these compound could also produce effects in the presence of PPARγ modulators. To begin this investigation we assessed the extent to which the PPARγ agonist Troglitazone and antagonist T0070907 affect hMSC differentiation. We also included GM6001 as a positive control. Since T0070907 has an apparent IC_50_ of ~1 nM [36], which is comparable to that of GM6001 and YHJ-7-52 for various MMPs, the final concentration used for these studies remained at 10 *μ*M. Human MSCs were induced toward an adipogenic lineage for 21 days in the presence of either YHJ-7-52, YHJ-7-82, GM6001, T0070907, or Troglitazone. Treatment with T0070907 reduced lipid accumulation by 20%, similarly to that of YHJ-7-52 and GM6001, while Troglitazone increased lipid accumulation by approximately 80% (Fig 3).

**Fig 4:**
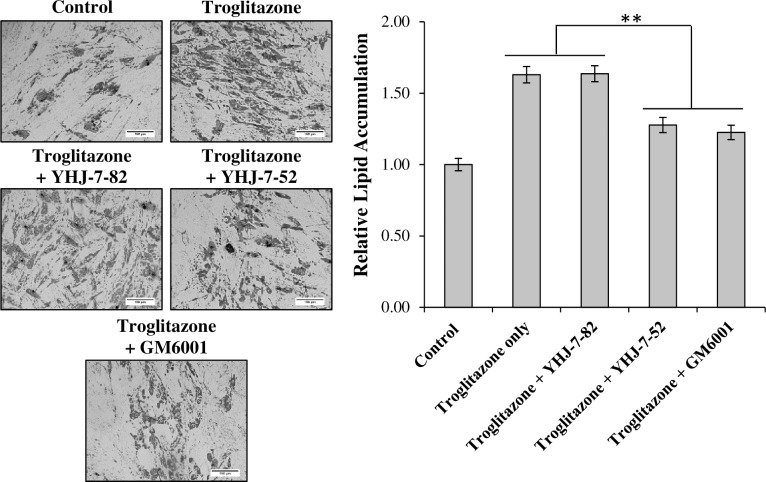
Co-treatment of differentiating hMSCs with Troglitazone and MMPIs. Cells undergoing differentiation in the presence of 10 *μ*M Troglitazone were co-treated with either 10 *μ*M YHJ-7-82, YHJ-7-52, or GM6001. DMSO vehicle was maintained consistent between treatments and used as control. Results are displayed as mean ± standard error. Scale bar 150 μm. ** *P* < 0.05.

We next determined if co-treatment with MMPIs could reverse the increases of lipid accumulation simulated by Troglitazone. Fig 4 shows that while all treatment conditions displayed substantially more lipid accumulation than untreated controls, the addition of YHJ-7-52 and GM6001 significantly reduced the ability of Troglitazone to increase lipid accumulation (22% and 25% reduction respectively).

**Fig 5:**
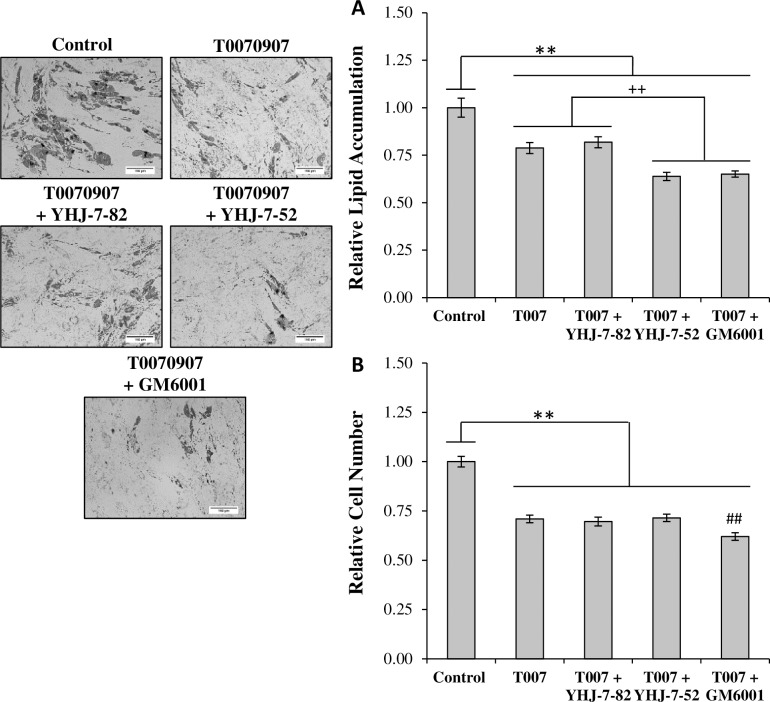
Co-treatment of differentiating hMSCs with T0070907 and MMPIs. (A) Cells undergoing differentiation in the presence of 10 *μ*M T0070907 were co-treated with either 10 *μ*M YHJ-7-82, YHJ-7-52, or GM6001. DMSO vehicle was maintained consistent between treatments and used as control. (B) Relative cell number was determined via nuclei counts. Results are displayed as mean ± standard error. Scale bar 150 μm. ** *P* < 0.05, ++ *P* < 0.05, ## *P* < 0.05 compared to T0070907.

Finally, since MMPIs and T0070907 operated upon different targets, the ability of T0070907 to work synergistically with an MMPI when reducing adipogenesis was investigated (Fig 5A). When treated simultaneously with T0070907, both YHJ-7-52 and GM6001 further reduced lipid accumulation by ~20%. YHJ-7-82 did not provide any additional reductions consistent with previous results. Additionally, while treatment with T0070907 reduced relative cell number by approximately 30%, co-treatment with YHJ-7-52 did not result in further cell number reductions (Fig 5B).

## Discussion

### YHJ-7-52 affects adipogenesis possibly through the modulation of PPARγ mRNA expression

It was established early on that adipogenesis is highly influenced by the mechanical stresses imposed by extracellular matrices [8, 37–40]. Modulation of the ECM during adipogenesis is suggested to influence cytoskeletal organization, which in turn can affect adipogenic signaling cascades, since the cytoskeleton provides a direct connection between the ECM and nuclear matrix [39, 41–48]. Furthermore, mechanical stress has been shown to affect the expression of PPARγ within stem cells undergoing adipogenesis [49]. Since, the primary means by which cells regulate ECM stiffness and composition is through the activity of MMPs, it was expected that YHJ-7-52 would affect PPARγ expression. Our qPCR results showed that YHJ-7-52 did indeed reduced PPARγ mRNA expression levels after days 9 of culture. However, complete suppression of PPARγ mRNA expression was not observed. Together with that fact that we observed a relatively similar reduction in lipid accumulation, it would indicate that MMPI treatment does not completely abolish adipogenic commitment within hMSCs, but it can influence the extent of differentiation.

### YHJ-7-52 and GM6001 display similar effects upon adipogenesis

During our earlier investigations with the YHJ series of thiol MMP inhibitors, we showed that while potent, they displayed lower levels of stability than hydroxamate compounds under standard enzyme inhibition assay conditions [26]. Despite having lower stability than the hydroxamate MMPI GM6001, YHJ-7-52 reduced lipid accumulation by similar amounts (Fig 3). One possibility is that the concentration used (10 *μ*M) is high enough to sustain the inhibitory activity for long enough periods of time to exert an influence. In addition, fetal bovine serum, which hMSCs require a greater percentage of, is composed of numerous factors including MMPs [50], thus a second possibility is that MMPs in the culture media, especially from fetal bovine serum, may bind to MMP inhibitors and prevent thiol oxidation and MMPI inactivation as discussed in our previous publication [26].

### MMP inhibition can still reduce lipid accumulation in the presence of a peroxisome proliferator activated receptor gamma (PPARγ) antagonist or agonist

Almost all previous studies investigating how MMPs affect obesity utilize either animal models or secondary metabolic markers in humans [33, 51–53]. However, numerous other factors can contribute to the outcome of these studies, such as angiogenesis [54] and macrophage activity [55]. As such, we decided to investigate the effects MMP inhibition had on overstimulation of hMSC adipogenesis with the PPARγ agonist Troglitazone. We observed that both YHJ-7-52 and GM6001 reduced Troglitazone-induced lipid accumulation both by approximately 25% (Fig 4). While future studies will need to investigate the effects of YHJ MMPI treatment on whole human fat tissues, our results indicate that MMP inhibition can, to an extent, reduce the effects of PPARγ overstimulation during adipogenic differentiation and suggest that these MMPIs could be used as molecular tools for adipogenesis and obesity treatment research.

Finally, co-treatment experiments were performed using a MMPI and a PPARγ antagonist T0070907 to determine if these compounds could work together in reducing lipid accumulation. We observed that YHJ-7-52 and T0070907 co-treatment produced an additive reduction in lipid accumulation, while no additional reduction in cell number was observed. This, implies that observed cell number reductions were due to T0070907. T0070907 is reported to be minimally cytotoxic up to 50 *μ*M [56] and treatments up to 20 *μ*M have reduced proliferation of MDA-MB-231 and MCF-7 breast cancer cells without induction of apoptosis [57]. As a result, it is likely the observed reductions in cell number from these experiments stem from T0070907 reducing proliferation, not apoptosis. Thus, it would appear that MMPIs can work in combination with other modulators of adipogenesis without substantially affecting cell number, an important consideration in light of the physiological significance of adipose tissue.

## Conclusions

Our results show that the novel MMPI, YHJ-7-52, is an effective regulator of hMSC adipogenesis. Moreover, our inhibitor reduced PPARγ mRNA expression, attenuating adipogenesis in hMSCs. MMP inhibition was also able to suppress lipid accumulation in adipocytes co-treated with either Troglitazone, an efficacious agonist of PPARγ, or with T0070907, a potent antagonist of PPARγ. Therefore, MMP inhibition provides a potential avenue for adipogenesis and obesity treatment research.
